# Buffalo Medical and Surgical Association

**Published:** 1884-09

**Authors:** Frederick Peterson

**Affiliations:** Secretary


### Buffalo Medical and Surgical Association.

*Regular Meeting, August 5, 1884.*

President, F. W. Bartlett, in the Chair.

The minutes of the last meeting were read and approved, after correction by Dr. Stockton, who stated that he would rather not be considered as thinking that obstetrics should be the work of the midwife.

Dr. Irving M. Snow was elected a member of the society.

Dr. A. R. Davidson read a paper on "Hæmaturia as a Symptom in Diseases of the Genito-Urinary System." (Published in the Journal for August.)

*Discussion*—Dr. Frederick thought every one would find difficulty in diagnosing the source of blood in the urine. It is almost the same as the differentiation of epithelium from the various portions of the genito-urinary tract.

Dr. Hartwig doubted if urine could be drawn from one ureter for purposes of careful diagnosis. One German author states that he has accomplished it. It is possible in the female, he thinks, by using the Siemens urethral dilator and the Siemens catheter for the ureter. He has himself tried it, once in the cadaver and once in a living person. He was quite successful in the dead body and introduced it two inches into the living ureter. Siemens has introduced it into the pelvis of the kidney itself. It is said that the openings of the ureters can be distinguished by feeling, but he had had difficulty in doing this.

Dr. Coakley thought the paper good as far as it went. The literature on this subject is not great. He wished more had been said of the treatment. He had met with more cases of renal difficulty in this city in three years than in Richmond in twenty, with twice as large a practice. He believed the cause must lie in the water we drink. The Niagara water contains more phosphates than most waters. Had noticed a periodicity in renal affections. They were numerous in the spring, ran through short courses and terminated favorably.

Dr. Hopkins knew what was meant by the parenchyma of the kidneys, but would like to know what was the signification of parenchyma of ureters and bladder?

Dr. Cronyn said the doctor had nicely summarized the subject of hæmaturia. Naturally, it is very important to know the origin of the blood which we find in the urine, in order to be able to treat the case properly, which is still more important. The therapeutics will be plain after the diagnosis. Reno-cystic disease prevails the world over, and he did not think it limited so much to Buffalo as Dr. Coakley seemed to believe. The literature of these diseases is vast in every language.

Dr. Cary was sure that renal affections prevail to a much greater extent in northern than in southern climates, and referred to the hospital statistics of Genoa, where occurred but one case in 3,000, while in London there was one in thirty.

Dr. Davidson thanked the gentlemen of the association for the kindly reception of the paper. He had feared that it might be considered as more appropriate for undergraduates than for a learned body of physicians of experience, but he had been induced to write the paper from his experience in many cases which had been referred to him, and particularly by the case of an eminent physician, who had consulted him with marked hæmaturia. In this case the patient had consulted a number of physicians, both here and in New York. He had had prescribed for him gallic acid, tannin, lead, ergot, tr. iron, etc.; no two physicians agreed upon the treatment, and the origin of the blood was only guessed at. To ask for the treatment of hæmaturia was like asking for the treatment of dropsy. The term designates a symptom only, and not a disease, and as the bleeding may originate from any part of the urinary tract, a vaguer term than hæmaturia can hardly be employed. He agreed, however, with Dr. Coakley, as to the prevalence of renal calculi in this region, and particularly those composed of oxalate of lime. He believed that the calcareous waters of this vicinity had an influence, and referred to the importance of prescribing in such cases the purer mineral waters, or, when the expense was an obstacle, the employment of water which had been boiled.

In reply to Dr. Hopkins' objection to the use of the word "parenchymatous," as applicable to the entire urinary tract—if he remembers correctly, the word "parenchyma" was made up of Greek words, signifying *to pour out*, and was particularly applied to the texture of the glandular organs, because it was believed that it was formed of effused blood.

In the paper, the term "parenchymatous hemorrhage" was used to signify an effusion or oozing of blood from any part of the urinary tract in contra-distinction to the term "profuse hemorrhage," by which was implied a rupture, however insignificant, of a vessel.

*Voluntary Communications*—Dr. Hayd exhibited the head and neck of a tænia mediocanellata. After using almost every remedy, he, at last, succeeded in bringing away the worm with a dose of pellitierin, the alkaloid of pomegranate. The patient had, at various times in the past few months, discharged pieces of the worm thirty, eighteen, twelve and twenty-eight feet in length, respectively.

Dr. Peterson asked if any of the members had met with the tænia solium, for all the heads sent to him for examination proved to be tænia mediocanellata. He would like to ask, further, if any gentleman present could affirm that he had seen a tape worm over sixteen feet in length, by his own actual measurement. The length given by German pathologists was not over sixteen feet.

Drs. Cronyn, Hayd and others were sure they had frequently seen them double that measurement. Dr. Cronyn said there were some in the museum at Toronto ninety feet in length. Dr. Hayd invited Dr. Peterson to come to his office and measure the one he had recently removed, which was thirty feet in length.

Dr. Davidson said that pomegranate had been tried by practitioners often, and, like most of the tænicides, with variable success in expelling the worm. Kooso was the best remedy for tænia, but the flowers must be perfectly fresh and the patient have abstained from food for at least twelve hours before taking the drug.

Dr. Hartwig had tried pellitierin three times. It was not only successful, but an agreeable drug to take.

Dr. Cronyn believed in the idiosyncrasies of people with regard to the effect of the various anthelmintics. Sometimes one remedy succeeds, sometimes another, owing to the difference of the person in susceptibility. He knew of one case cured by fasting and praying.

Dr. Hayd exhibited, also, a one-month ovum with the membranes complete.

Dr. Frank related the case of a man aged 62, ship-carpenter, who came to him two weeks ago, because of his vomiting everything. From the great pulsation in the epigastric region and other signs, he made a diagnosis of an abdominal aneurism, but found no aneurism at the autopsy. The heart was enlarged and there was some calcification of the abdominal aorta. On opening the stomach, which was greatly distended, he found an organized clot of blood as large as a fœtal head. The day before he died, the patient had vomited blood. He would like to ask how long this clot could have been in the stomach and where the blood came from, as the stomach seemed normal?

Dr. Park said there were often cases which simulated abdominal aneurism. He had seen two or three. The chief pathognomonic signs were the intense pain experienced in aneurism here, when pressure is made upon the iliacs, and the sensation of lateral expansion.

Dr. Peterson doubted if a clot could organize in the stomach, because of the presence of the gastric juice, and believed the blood had been in the stomach but a few hours, if so long. It evidently came from the rupture of a minute vessel in the gastric wall, which is not a very uncommon occurrence.

*Reports on Prevailing Diseases*—Dr. Weil reported dysentery; Dr. Hartwig, the same, and various forms of choleraic disease; Dr. Coakley, cholera infantum and morbus; Dr. Van Peyma, the same; Dr. Ring, the same, with dysentery; Dr. Cronyn, diarrhœa and dysentery, and Dr. Bartlett stated that he had not, in thirty years of practice, witnessed so much intestinal trouble, such as cholera infantum and morbus, diarrhœas and dysentery.

*Miscellaneous Business*—The following resolution was introduced, and after some discussion adopted, the Secretary being directed to send copies of the same to the leading newspapers:

WHEREAS, We feel that the pangs of private sorrow are not lightened by the parading of its details in the public press, and that such publicity, while at the cost of additional suffering to those already severely tried, merely panders to the vulgar and debasing desire for the perusal of personal items.

WHEREAS, The *Buffalo Morning Express*, of August 3d, contained an article under the heading of "Two Unfortunates," which called public attention to the private afflictions of two families of this city in a manner to cause added and unnecessary grief to worthy and innocent people.

WHEREAS, In sending our patients to institutions, where, from the nature of their cases, they are better cared for than at home, we still regard them in the light of private persons whose unhappy ailments are no more to be dragged before the public than are ordinary professional confidences; therefore—

*Resolved*, That the Buffalo Medical and Surgical Association censures the publication in the *Express* as an inexcusable, wanton and brutal invasion of the rights of the individual and of the home.

The meeting then adjourned.

FREDERICK PETERSON, *Secretary*.

